# Redesigning the Hospital Environment to Improve Restfulness

**DOI:** 10.1001/jamanetworkopen.2024.47790

**Published:** 2024-12-04

**Authors:** Caellagh D. Catley, Kayla Paynter, Kendall Jackson, Ashley Huggins, Jenny Ji, Sai Anusha Sanka, Michelle Simkins, Thomas M. Maddox, Patrick G. Lyons

**Affiliations:** 1 Washington University in St Louis School of Medicine, St Louis, Missouri; 2 Healthcare Innovation Lab, BJC HealthCare, St Louis, Missouri; 3 Center for Clinical Excellence, BJC HealthCare, St Louis, Missouri; 4 Division of Cardiology, Washington University School of Medicine, St Louis, Missouri; 5 Division of Pulmonary and Critical Care Medicine, Washington University in St Louis School of Medicine, St Louis, Missouri; 6 Now with Oregon Health & Science University, Portland

## Abstract

**Importance:**

Hospital wards are often not conducive to patient sleep, negatively affecting patient health and experience.

**Objectives:**

To assess determinants of in-hospital restfulness and to design and test rest-promoting interventions on the wards in partnership with clinicians, staff, and patients.

**Design, Setting, and Participants:**

This rapid-sequential mixed-methods quality improvement study was performed at a large urban academic hospital in St Louis, Missouri, from May 1, 2021, to December 31, 2022, with follow-up through hospitalization. Mixed-methods activities involved purposively selected hospitalized adults on the wards, nurses, hospitalists, and hospital staff. Interventions included all adult hospitalizations on the study wards.

**Interventions:**

Sequential stacked bundles of multimodal rest-promoting interventions (general education, focused education on light or noise, safely reducing overnight monitoring or testing, and environmental personalization).

**Main Outcomes and Measurements:**

Pre-post comparisons of perceived nighttime quietness (via surveys) and sleep opportunity (coprimary outcomes) as well as clinical interruptions overnight, environmental noise, and adoption and satisfaction.

**Results:**

Nine patients (4 female) and 14 staff members (10 female; 3 nurse managers or administrators, 10 nurses, and 1 physician) were interviewed, 38 surveys were collected, and more than 100 hours of observation were performed. Interventions were evaluated for 671 patients (mean [SD] age, 60 [16] years; 336 [50%] female). Determinants of in-hospital rest included infrastructure, staff attitudes, priorities, culture, and patient experiences of anxiety, uncertainty, and loss of control. Informed by these determinants, codesign workshops yielded 39 potential interventions, from which 9 were selected for testing. Related interventions were organized into bundles, which were tested in sequential 2-week sprints. Perceived nighttime quietness improved nonsignificantly during the project (wards “always” quiet at night: 51% preintervention vs 86% postintervention; *P* = .09), with excessive noise events decreasing from 0.65 (95% CI, 0.53-0.77) to 0 per 100 patient-nights before the intervention (*P* = .02). Sleep opportunity improved significantly (mean, 4.94 [95% CI, 4.82-5.06] hours per patient-night before the intervention vs 5.10 [95% CI, 5.00-5.20] hours per patient-night after the intervention; *P* = .01).

**Conclusions and Relevance:**

In this quality improvement study, a set of feasible, acceptable, and beneficial rest-promoting interventions were developed. After implementation of these interventions on the wards, a significant improvement in nighttime quietness and sleep opportunity was observed. These results demonstrate how human-centered design methods can generate practical and effective strategies for improving an important patient-related outcome and a core element of patient experience.

## Key Points

**Question:**

What determines sleep and rest on inpatient wards, and can these determinants inform the design of effective rest-promoting interventions?

**Findings:**

This quality improvement study at a single hospital elicited novel insights regarding the landscape of rest in the hospital. These insights were used to create and implement a set of feasible and acceptable rest-promoting interventions that were associated with improved nighttime quietness and sleep opportunity.

**Meaning:**

This work underscores the value of an emerging set of methods for patient-centered problem-solving and introduces new avenues for future intervention development, including rest-promoting activities outside nighttime hours and increased focus on personalization.

## Teaser

This quality improvement study examines determinants of in-hospital restfulness and tests rest-promoting interventions in the ward environment.

## Introduction

Inpatient wards are notoriously disruptive environments for patients. Nighttime disturbances are particularly common, reducing sleep by more than an hour per night for most patients.^1,2^ Overnight disturbances are often caused by pain, excessive light and noise, nursing assessments, and awakenings for blood draws and medication administration.^1,3,4,5,6,7,8,9,10^ Many disturbances are worsened on high-acuity wards because of increased monitoring and clinical activity in multioccupancy rooms.^4^

In addition to their detrimental contributions to the patient experience,^11^ these disruptions carry negative health consequences for many patients. Sleep fragmentation and deprivation exacerbate circadian dysregulation and delirium, often inducing a vicious cycle in which progressive daytime fatigue and inactivity yield paradoxically increased nocturnal restlessness. Poor hospital sleep has been associated with cardiovascular and metabolic disorders, impaired wound healing, and posthospital syndrome.^12,13,14,15,16^

Although rest is increasingly recognized as important for hospitalized patients, including several studies evaluating interventions to improve restfulness in the hospital, poor sleep in the inpatient setting remains an incompletely addressed challenge.^17,18,19,20,21^ To date, most efforts to measure and improve sleep in the hospital have been unsuccessful, been limited to specific populations, or involved large system-level interventions in strictly controlled settings, which may be impractical in many clinical environments.^22^ To address this challenge at our hospital, we partnered with clinicians, staff, and patients to examine determinants of in-hospital restfulness and to iteratively prototype and test rest-promoting interventions in the ward environment. We aimed to identify barriers to patient rest in the inpatient setting, design and implement solutions to this problem, and measure implementation and process outcomes.

## Methods

### Study Design, Setting, and Participants

The Healthcare Innovation Lab—a team of clinician leaders, applied researchers, and project specialists—serves BJC HealthCare (a large integrated system in St Louis, Missouri) and Washington University School of Medicine by catalyzing, testing, and implementing new approaches to care delivery.^23,24,25,26,27^ In 2021, at the request of leaders from Barnes-Jewish Hospital (an urban academic hospital), we sought to improve the patient experience of restfulness on the hospital wards, which had been identified as an opportunity based on findings from Hospital Consumer Assessment of Healthcare Providers and Systems (HCAHPS) surveys.^11^ Washington University’s Institutional Review Board approved the prototyping and testing activities (exploratory data collection was designated quality improvement and thus not subject to approval). Verbal consent was obtained from participants for prototyping. We followed the Standards for Quality Improvement Reporting Excellence (SQUIRE) reporting guideline in conducting and reporting this project^28^; the eMethods in Supplement 1 contain further details.

We conducted a quality improvement study of stacked rest-promoting sleep interventions on a medical-surgical ward at Barnes-Jewish Hospital from May 1, 2021, to December 31, 2022. Because we expected the problem and its context to be complex and multifactorial (ie, some drivers of sleep limitations are environmental, whereas others are organic; only some environmental factors are modifiable),^11,29^ we used methods from human-centered design (HCD) to understand challenges and develop solutions informed by the experiences and needs of patients and their care teams. Subsequently, we implemented and tested solutions with pre-post comparisons.

Participants included patients (hospitalized adults who spent at least 1 night on the study ward), nurses, and hospitalist physicians. First, we invited hospitalized patients and their nurses to participate in the project’s formative stages. Second, we engaged bedside nurses, nurse leaders, and hospitalists for ideation and prototyping. Third, we evaluated nursing-, patient-, and ward-level outcomes during the intervention period. We used purposive sampling—considering role, time in role, day vs night shift, and gender—for all qualitative activities to increase diversity of participants’ experiences and perspectives. We did not collect data on participants’ race or ethnicity because no race-related research questions were posed and we sought to minimize participant discomfort regarding the potential perception that race could affect their care delivery behaviors.

### Theory and Frameworks

**Figure 1.**
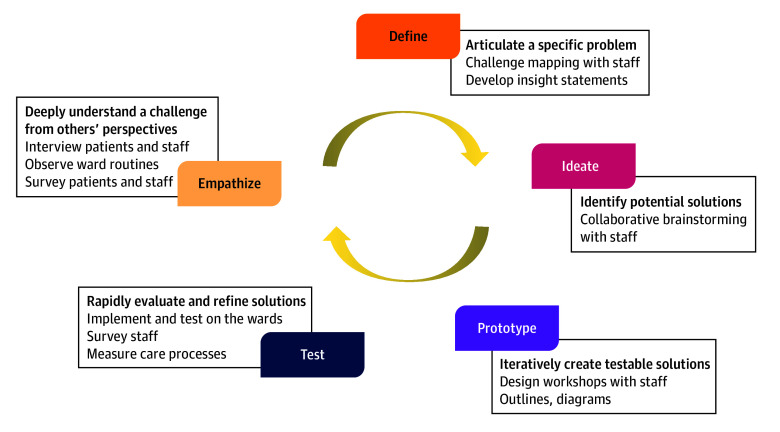
Key Human-Centered Design Activities During Formative Research

Human-centered design is an approach to problem-solving that prioritizes a deep understanding of participants’ experiences and needs, rapid iteration, and codesign to create appropriate solutions (Figure 1).^30,31,32^ On the basis of IDEO’s approach,^33^ we used HCD methods to understand and characterize specific challenges, mapping findings to a logic model (eFigure 1 in Supplement 1) and to constructs within the Consolidated Framework for Implementation Research (CFIR) to organize insights systematically.^34,35,36,37^ We then used HCD methods to design and evaluate possible solutions.

### Formative Research: Empathy and Problem-Defining

From May 1, 2021, to June 30, 2021, we used rapid exploratory sequential mixed methods to examine determinants of rest on 2 standard medical-surgical wards.^38,39^ Semistructured interviews (eMethods in Supplement 1) explored patient characterizations of rest in the hospital, patient and nurse perceptions of hospital daytime and nighttime routines, and determinants of these.^40,41^ We directly observed the study ward for approximately 100 hours for 11 visits, taking detailed field notes on overnight activity with particular attention to noise, light, interruptions, and factors emerging from interviews. We triangulated qualitatively derived insights against patients’ perceived sleep quality (quantified via the Verran and Snyder Halpern Sleep Scale])^42^ and ward-level summaries of electronic health record (EHR) data to characterize the timing of vital sign measurement, blood work, and medication administration. These data informed interactive challenge mapping (structured exercises and questions to clarify and conceptualize complex problems before solution development) with a multidisciplinary group of hospital staff.^43^

### Intervention Development and Evaluation: Ideating, Prototyping, and Testing Solutions

We led nurses and nurse leaders through 2 design workshops to ideate solutions and outline prototypes (COVID-19–related constraints prevented including patients in this step). Participants conceptualized potential interventions through structured exercises.^44^ In small groups, participants then codeveloped hypothetical rest-improving intervention bundles linked by thematic similarity and expected resource requirements. We consolidated these products into a final series of interventions for testing.

**Figure 2.**
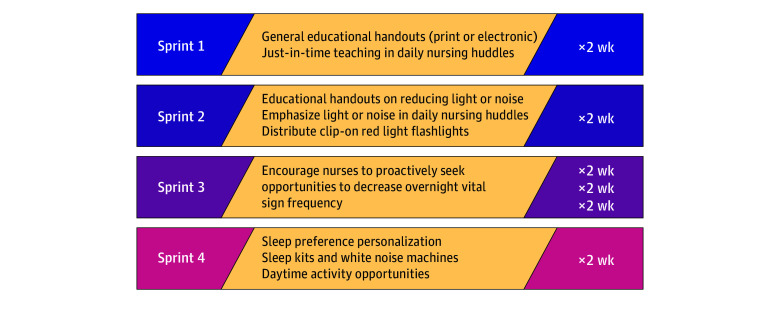
Intervention Chronology

We tested interventions in an iterative series of Agile Innovation sprints on the study ward that had most engaged with formative work (Figure 2).^45^ After gathering baseline data, we introduced interventions in 2-week increments followed by 2-week breaks (for sufficient HCAHPS data collection), refining subsequent interventions based on the most successful elements in each sprint. A priori, we sequenced interventions from least to most intensive in terms of materials and participant effort such that each sprint could build on prior weeks’ successful practices. During the project, we modified our initial plan for 4 discrete intervention sprints; to validate early promising findings in the third sprint, we iterated this intervention for an additional 4 weeks before introducing the final intervention.

### Outcomes

The coprimary outcomes were sleep opportunity (maximum time between overnight clinical interruptions identified in the EHR)^21^ and patient perceptions of nighttime noise (percentage reporting that the wards were “always quiet” on routine HCAHPS surveys).^46^ We also measured clinical interruptions overnight (by EHR data), environmental noise (Yacker Tracker, AGI Attention Getters Inc), and staff perceptions of adoption (by nurse manager report) and satisfaction (by survey). We report continuous data as mean (SD) and categorical data as number (percentage).

### Statistical Analysis

We compared primary outcomes between the preintervention period and the final 2-week intervention sprint. We compared secondary outcomes between the preintervention period and each sprint. We used 2-tailed, unpaired *t* tests (continuous data), χ^2^ tests (categorical data), and analysis of variance with post hoc Tukey tests (longitudinal comparisons across sprints) for all comparisons. We used SAS Enterprise Guide, version 7.15 (SAS Institute Inc) for all analyses and considered a 2-sided *P* ≤ .05 to be statistically significant.

## Results

### Empathy, Problem Identification, and Ideation

We interviewed 9 patients (4 female and 5 male) and 14 staff members (10 female; 3 unit nurse managers or administrators [2 night shift], 10 nurses [4 day shift, 5 night shift, and 1 variable shifts], and 1 hospitalist physician [variable shifts]), collected 38 surveys, and performed more than 100 hours of direct observation. Rapid qualitative analysis highlighted that patients valued rest (restorative periods of relaxation and recovery, even while awake) in addition to sleep. We therefore identified determinants of effective rest in the hospital across CFIR domains, primarily under the inner setting, outer setting, and individuals domains (eTable 1 in Supplement 1).

In terms of the inner setting, patients and nurses related how physical, information technology, and work infrastructure converged to hinder nighttime sleep opportunities. Physical environment determinants included noise (eg, from roommates or construction), intrusive lighting, and unsatisfactory control over ambient temperature. Disruptive beeping from intravenous infusion pumps mapped to the information technology construct because multiple nurses hypothesized that remotely alarming pumps (eg, notifying staff via wireless communication rather than audibly alerting in the room)^47^ might reduce noise substantially. Work infrastructure included behavioral and workflow-driven interruptions limiting sleep opportunity, such as overnight vital sign measurements and laboratory test sample draws as well as repeated early morning awakenings for rounding activities. Relatedly, nurses described the prevailing attitude that “ultimately, the care that you need is a higher priority than sleep,” reflecting the perception that some elements of overnight care are potentially incompatible with—and should take precedence over—optimal rest.

Some of these determinants were also closely related to local attitudes, external pressures, and policies from the outer setting. Specifically, nurses described pressures related to safety culture and role expectations (eg, sleep interruptions in service of task completion and thorough handoff communications to the next shift) as barriers to rest.

Within the individuals domain, patients described situational determinants of rest, including anxiety, clinical uncertainty, and loss of control. Some reported limited restfulness even during times with relatively few interruptions, linking boredom and loneliness to uncertainty and anxiety. For these patients, baseline social and financial stressors were compounded by new medical stressors and limited control over these. Finally, we observed variation in how patients experienced rest in the hospital. Although many patients expressed frustration with sleep disruption on the wards, some patients (including several with housing instability) described the hospital as safer and more comfortable than their baseline environment.

On the basis of these determinants, we collaborated with key informants to develop a challenge map (eFigure 2 in Supplement 1) and insight statements to guide ideation sessions for potential solutions. These guiding statements were (1) focus on rest, not just sleep; (2) let patients control their environment; and (3) every patient is different.

### Interventions

Ideation sessions yielded 39 unique potential interventions corresponding to CFIR domains, which we organized in an impact-effort prioritization matrix (Table 1) to optimize resource allocation and expected value.^48,49^ We selected 9 items for testing through collaborative discussion with ward staff (Table 2 and Figure 2), grouping them into bundles according to expected workload intensity and resource needs based on feedback from the advisory groups and nursing management teams on the study ward (eFigure 3 in Supplement 1). We tested interventions in discrete 2-week sprints during a period of 6 months, beginning with interventions that could be quickly implemented and would minimally disrupt clinical workflows.

**Table 1.** Impact-Effort Matrix for All Brainstormed Intervention Ideas

Impact	Effort		
	Low	Moderate	High
High	Preference checklist: temperature, light, routine; explicit quiet hours (signage and expectations); daily patient routine templates (eg, on whiteboard in room); white noise machines available for each room	Sleep-promoting EHR order defaults (eg, routine medication start times, overall and nighttime vital sign frequency, timing of routine laboratory test sample draws)	“Smarter” intravenous pumps; more comfortable beds; adjustable beds; higher-quality pillows; higher-quality curtains; higher-quality room dividers; daily “turndown service”/sleep concierge; increased recreational therapist presence; smart home hospital rooms; restricted times for rounding by policy; restricted times for routine laboratory test sample draws and tests by policy; restricted times for routine medications by policy; elimination of double-occupancy rooms; more staffing
Moderate	Increased activity cart availability; check for lights off at bedtime; check for closed door at bedtime; clip-on red light flashlights; sleep kits with eye mask and ear plugs; fans available for each patient; increased blanket availability; default use of television timers; ward-level decibel tracker competition; patient education: sleep importance, available options and interventions	Routine measurement and reporting of ward-level rates of abnormal nocturnal vital signs	Staff education on value of sleep; better handoff: education on medications and practices; more comfortable gowns (including better ease with wires and lines); more comfortable linens; sleep on problem list: standard ask; routine therapy animal visits
Low	Daily newspaper availability; default soothing television channel; meditation mobile application; nightly request for telephones on vibrate; care team biographical information handouts	None	Aromatherapy

Abbreviation: EHR, electronic health record.

**Table 2.** Bundle Components

Sprint No.	Interventions	Intervention target	Relevant measurements
1 (General education)	Electronic educational handouts; print educational handouts; just-in-time teaching in nursing daily huddles	Individuals (intervention deliverers); inner setting (work infrastructure, compatibility, relative priority, and mission alignment); outer setting (local attitudes)	Sleep opportunity; nightly interruptions; noise; patient satisfaction; staff satisfaction
2 (Specific education)	Educational handouts on reducing light and noise; just-in-time emphasis on reducing light and noise in daily nursing huddles; red light clip-on flashlights	Individuals (intervention deliverers); inner setting (work infrastructure, compatibility, relative priority, mission alignment, and available resources)	Sleep opportunity; nightly interruptions; noise; patient satisfaction; staff satisfaction; adoption of practice change
3 (Reduce interruptions)	Encourage nurses to proactively seek opportunities to decrease overnight vital sign frequency	Individuals (intervention deliverers); inner setting (work infrastructure)	Sleep opportunity; nightly interruption; patient satisfaction; staff satisfaction; adoption of practice change
4 (Personalization)	Sleep preference personalization checklist; sleep kits and white noise machines; daytime activity opportunities (eg, activity cart and newspapers)	Individuals (intervention recipients); inner setting (work infrastructure and physical infrastructure)	Sleep opportunity; nightly interruptions; patient satisfaction; staff satisfaction; adoption of personalization template in patient care

In the first intervention, we distributed a 1-page educational document (eMethods in Supplement 1) by email and in print (at each nursing workstation). We paired this material with brief informal educational sessions presented each weekday by study team members at preshift nursing huddles. The second intervention was similar but focused on reducing (1) unnecessary nighttime interruptions via planned clustering of care and (2) nocturnal light and noise; we distributed red-wavelength clip-on flashlights to night-shift nurses with guidance for using them instead of overhead lights in patient rooms. In the third intervention, we encouraged staff to minimize unnecessary nighttime interruptions, at their discretion, for patients deemed stable. We repeated this step 2 additional times to validate improvement on interim measurements of nighttime disruptions and to ensure that appropriate discretion was being used to decrease interruptions. The fourth intervention emphasized personalization of the patient rest environment, including encouraging nurses to use a novel sleep preference prioritization tool (eMethods in Supplement 1) in their daily patient assessments, distributing sleep kits and white noise machines to all patients, and providing activities for patients during the day.

### Evaluation

Interventions were evaluated for 671 patients (mean [SD] age, 60 [16] years; 336 [50%] female and 335 [50%] male). The preintervention period encompassed 981 patient-nights from 217 unique patients, while the intervention periods involved a total of 2028 patient-nights (266 patients-nights in intervention 1, 328 in intervention 2, 1179 in intervention 3 [371 followed by 373 and 453], and 237 in intervention 4). Patient characteristics are given in eTable 2 in Supplement 1. Among HCAHPS respondents (n = 69), perceived nighttime quietness improved nonsignificantly during the study period (Table 3; eTable 3 in Supplement 1). Compared with the preintervention period (51% [24 of 47]), patients more frequently rated their hospital ward as “always quiet” after the third (10 of 15 [67%]; χ^2^ = 1.12; *P* = .29) and final (6 of 7 [86%]; χ^2^ = 2.96; *P* = .09) interventions. Correspondingly, excessive noise events decreased from 0.65 (95% CI. 0.53-0.77) to 0 per 100 patient-nights preintervention after the first and all subsequent intervention sprints (*P* = .02).

**Table 3.** Quantitative Outcome Measures

Measure	Preintervention (n = 981 patients-nights and 217 patients)	Poststudy (n = 237 patient-nights and 65 patients)	*P* value
Coprimary outcomes			
Patients rating the hospital wards as “always quiet,” total No./No. (%)^a^	24/47 (51)	6/7 (86)	.09
Sleep opportunity per patient-night, mean (SD), h^b^	4.94 (1.86)	5.10 (1.65)	.01
Secondary outcomes			
Clinical interruptions per patient-night, mean (SD)^c^	2.69 (1.95)	2.78 (2.02)	.09
Excessive noise events per 100 patient-nights, mean (SD)^d^	0.65 (1.3)	0	.02

Abbreviation: HCAHPS, Hospital Consumer Assessment of Healthcare Providers and Systems.

^a^ Calculated as number (percentage) rating hospital wards as “always quiet” on posthospitalization HCAHPS.

^b^ Calculated as longest time between electronic health record–measured interruptions from 10 pm to 6 am.

^c^ Calculated as unique recordings of blood pressure, fingerstick blood glucose, laboratory blood tests, and scheduled medication administrations.

^d^ Calculated as number of recordings greater than 35 dB per night.

Sleep opportunity increased significantly during the project. Mean sleep opportunity was 4.94 (95% CI, 4.82-5.06) hours per patient-night in the preintervention period and 5.10 (95% CI, 5.00-5.20) hours after the final intervention (*P* = .01), corresponding to a mean increase of 9.6 (95% CI, 0.24-19.0) minutes in sleep opportunity. Clinical interruptions did not substantially change during the project, although overnight blood pressure measurements became significantly less frequent during the intervention period (eTable 4 in Supplement 1).

We observed high levels of adoption and satisfaction for all interventions. Mean self-reported adoption (“During your shifts, how often did you…?”) was 4.50 (95%, CI, 4.21-4.79; 12 responses) on a 5-point scale, with 5 representing “every patient, every shift.” Among 28 night-shift nurse responses (from 48 invitations), mean satisfaction was 4.60 (95% CI, 4.39-4.81). Informal feedback from nursing staff was uniformly positive.

## Discussion

Using HCD methods, we successfully codesigned a set of feasible, acceptable, and beneficial rest-promoting interventions alongside ward nurses at our hospital. Sleep opportunity and overnight noise improved significantly after the interventions, coinciding with nonsignificant increases in perceived nighttime quietness among the subset of patients completing posthospitalization surveys. Nurses rated these interventions as highly satisfactory, and informal evidence of adoption was strong. These results demonstrate how HCD methods can generate practical and effective strategies for improving an important patient-related outcome and a core element of patient experience.^50,51^ The degree of improvement in sleep opportunity and nighttime quietness is particularly notable because these are multifactorial measures, partially depending on issues such as staffing and local construction noise, that did not change during the project.^52^

We observed statistical improvement in sleep opportunity but no major change to nighttime interruptions with these interventions. It is possible that differential interruption cadence after the intervention enabled slightly longer periods of sleep opportunity. However, stability in the total number of interruptions also suggests that merely encouraging nurses to minimize interruptions is insufficient to change longstanding clinical practice; it remains unknown whether additional education and/or policy could address this issue. A second possibility is that some interruptions could not have been abrogated safely, such that patients might face a ceiling effect on sleep opportunity but still stand to benefit from minimizing disruptive factors (eg, noise and light) within time blocks with no clinical interruptions. We note that our sleep opportunity measurements are similar in magnitude to those reported previously,^21^ which raises the possibility of limited room for further improvement in uninterrupted time overnight.^53^ Finally, improved sleep opportunity guarantees neither actual patient sleep (which could change in duration, quality, or both within a given window of opportunity) nor patient perceptions of rest; future work should endeavor to measure these outcomes.

This project illustrates specific ways in which HCD methods add rigor and value to quality improvement efforts in the hospital. First, HCD’s foundational emphasis on empathy enables targeting the right problem and prioritizing relevant and acceptable solutions. In this case, patients emphasized to us that rest, even beyond sleep, is lacking in the hospital; limited sleep on the wards is often compounded by the psychological and emotional weight of illness, disrupted routines, loss of control, limited activity, and boredom. Recognizing that these challenges may play underappreciated roles in healing and recovery by increasing stress, anxiety, and the likelihood of further sleep impairments,^54^ we focused certain intervention components on opportunities not specifically related to sleep, including enhanced daytime recreational activities and tools to improve patients’ control over their environment regardless of time of day. Because conflating sleep and rest will have important implications for intervention design and evaluation, future work in this area should endeavor to disentangle these concepts as much as possible. Additionally, codesign and rapid prototyping increased the degree to which solutions were tuned to patient needs, staff workflows, and overall practicality. Improving both subjective and objective outcomes in several domains quickly and without modifying organizational determinants (eg, policy and procedures) underscores this value and raises the possibility that further gains could be made through higher-intensity interventions within the inner and outer settings.

Second, further underscoring HCD’s value is a final key learning from our inquiry: patient agency is perceived as an important determinant of rest, especially amid heterogeneous social situations, past experiences, and preferences. Facing clinical uncertainty as well as unpredictable—and often inconvenient—timing of rounds, tests, and results, many patients described their hope for increased control over their physical environment and routines. More than 1 interviewee described how personally tailored environments might make their evenings “more like home.” Although our final intervention illustrates one approach toward addressing this need, future work should consider other modalities of patient-centered personalization.

Our findings compare favorably with prior work in this area. For instance, we found similar magnitudes of sleep opportunity to those reported from a randomized clinical trial,^21^ lending validity to the obtainability of this measurement in clinical data. Future work should study whether sleep opportunity has value as a quality performance indicator for local or external benchmarking, since sleep can be interrupted by activities that are not recorded in the EHR. Our results also underscore others’ conclusions that even modest changes to clinical environments and routines may improve the inpatient rest experience while highlighting this area as an opportunity for potential further improvements in care quality and patient outcomes.^29^ Finally, this work builds on past literature demonstrating nursing-led interventions as potential avenues to decrease rest disruption in the hospital.^4,55^ Important future directions for this work include directly measuring sleep duration and quality through patient report and wearable technology, measuring clinical, safety, and cost outcomes, and evaluating sustainability and scalability.

### Strengths and Limitations

Our work is notable for several strengths. First, the methodologic rigor of this project’s formative stages allowed us to glean deep insights about patient needs, staff priorities, and clinical workflows while building rapport and empathy with our stakeholders. Second, our HCD-based approach to problem identification, ideation, and intervention development yielded highly feasible, easily implementable, and patient-centered solutions that we successfully deployed during a relatively short time horizon. Third, our focus on pragmatism and flexibility, including rapid qualitative methods, just-in-time modifications to intervention sprints, and diverse outcome measures, allowed us to complete all phases of the project efficiently while capturing meaningful patient, process, and system outcomes. Pragmatism and flexibility were essential in the face of COVID-19–related constraints, including restrictions on some patient-facing research activities, staffing shortages, and competing institutional priorities. Although these constraints limited our ability to include patients in design activities, obtain more systematic measures of adoption, or develop more intensive interventions (eg, remote intravenous pump notifications and EHR modifications) within the project’s timeframe, they also encouraged creativity and practicality.^56^

Our work also has several limitations. First, our pre-post study design leaves these findings vulnerable to the Hawthorne effect, regression to the mean for a ward selected on poor baseline quietness, and potential bias from intervention fatigue or unmeasured interventions (eg, although hospital construction was ongoing throughout the project, it is possible that the type or relative location of evening construction activities had changed). Second, limited HCAHPS completion raises the possibility of response bias for an outcome already limited by small sample size. Third, although we did not specifically measure safety outcomes, our longitudinal informal engagement with ward staff did not reveal concerns over delayed or missed recognition of clinical deterioration. Fourth, we were unable to include patients directly in design ideation and prototyping activities; however, in these sessions, we explicitly prioritized the challenges and potential solutions most frequently emphasized by patients during interviews and fieldwork. Fifth, our findings so far represent only the peri-intervention period on a highly engaged hospital ward, which may limit generalizability and which cannot yet offer information regarding long-term sustainability (although successfully stacking interventions over 6 months indicates sustainability in the near term).

## Conclusions

In this quality improvement study, we successfully codesigned a set of feasible, acceptable, and beneficial rest-promoting interventions alongside patients and staff at our hospital. After implementing these interventions on the wards, we observed substantial improvement in a key measure of patient experience. These results represent an important demonstration of how HCD methods can generate practical and effective strategies for improving an important patient-related outcome and a core element of patient experience.

## Notes

Supplement 1. **eMethods.** Expanded Methodology **eFigure 1.** Logic Model **eFigure 2.** Challenge Map **eFigure 3.** Intervention Bundle Concepts **eTable 1.** Impact-Effort Matrix for All Brainstormed Intervention Ideas **eTable 2.** Patient Characteristics and Ward-Level Outcomes **eTable 3.** Study Outcomes Across Interventions **eTable 4.** Clinical Interruptions Across Interventions

Supplement 2.
Data Sharing Statement
