# Supplementary material for: Redesigning the Hospital Environment to Improve Restfulness
Source: JAMA Netw Open. 2024 Dec 4;7(12):e2447790. doi: 10.1001/jamanetworkopen.2024.47790 (PMC11618460; doi:10.1001/jamanetworkopen.2024.47790)
Supplement: Supplement 1. — eMethods. Expanded Methodology eFigure 1. Logic Model eFigure 2. Challenge Map eFigure 3. Intervention Bundle Concepts eTable 1. Impact-Effort Matrix for All Brainstormed Intervention Ideas eTable 2. Patient Characteristics and Ward-Level Outcomes eTable 3. Study Outcomes Across Interventions eTable 4. Clinical Interruptions Across Interventions [file jamanetwopen-e2447790-s001.pdf]

Supplementary Online Content

Catley CD, Paynter K, Jackson K, et al. Redesigning the hospital environment to improve restfulness. *JAMA Netw Open*. 2024;7(12):e2447790.
doi:10.1001/jamanetworkopen.2024.47790

eMethods. Expanded Methodology

eFigure 1. Logic Model

eFigure 2. Challenge Map

eFigure 3. Intervention Bundle Concepts

eTable 1. Impact-Effort Matrix for All Brainstormed Intervention Ideas

eTable 2. Patient Characteristics and Ward-Level Outcomes

eTable 3. Study Outcomes Across Interventions

eTable 4. Clinical Interruptions Across Interventions

This supplementary material has been provided by the authors to give readers additional information about their work.

© 2024 Catley CD, et al. *JAMA Network Open.*

eMethods. Expanded Methodology

Study Design and Setting

We selected the study wards based on low pre-project performance on sleep-related domains of the Hospital Consumer Assessment of Healthcare Providers and Systems (HCAHPS) surveys, as well as high levels of clinical staff engagement during early formative work.

Theory and Frameworks

HCD is an iterative approach to problem-solving that prioritizes a deep understanding of participants' experiences and needs, rapid iteration, and co-design to optimize creation of appropriate solutions.[30,31] Key pillars of HCD are empathy (understanding stakeholders' experiences and needs), defining a problem, ideating potential solutions with participants, prototyping these solutions, and testing prototypes in low- and high-fidelity scenarios.[32] We used HCD methods to understand and characterize specific challenges, mapping findings to a logic model and to domains and constructs within the Consolidated Framework for Implementation Research (CFIR, a theory-informed determinants framework) to systematically organize new insights.[33–36] We then used HCD methods to design and evaluate possible solutions. The CFIR can inform the design and tailoring of interventions and implementation strategies through systematic assessment of barriers, facilitators, and contextual features (e.g., characteristics of complex settings or social structures) necessary for successful adoption and use of design products.[1,2] HCD methods can be paired with the CFIR for the purposes of co-design,[3] but this approach is relatively new.

The CFIR guided survey design and preparation of interview guides. This framework comprises five domains: intervention characteristics, outer setting (e.g., health care system policies), inner setting (e.g., facility characteristics, local culture), characteristics of individuals involved, and implementation processes (e.g., dissemination). We focused on the inner setting, outer setting, and individual characteristics domains.

© 2024 Catley CD, et al. *JAMA Network Open.*

Study Team

The project was designed and co-led by a project manager with training in Agile methodology (KP) and a physician-scientist with advanced training in HCD methods (PGL).[4,5] Project leaders trained 3 individuals (CC, KJ, AH) in rapid qualitative methodology including interviews and observations prior to study initiation. In addition to the authorship team, we also assembled a panel of subject matter experts and key informants to design and conduct this project. The team included representatives from Barnes Jewish Hospital's (BJH's) Patient Experience group, BJH's Division of Nursing Research, clinical-translational sleep researchers from Washington University, and hospitalist physicians and nursing staff from the wards. Longitudinally for the project's duration, this panel met regularly to consolidate observations and use these findings to inform subsequent phases of work.

Formative Research: Empathy and Problem Defining

From May 2021 to June 2021, we used rapid exploratory sequential mixed methods to identify and understand determinants of rest on the study ward.[37,38] Because empathy (understanding a problem through the perspectives of those most affected) is a hallmark of HCD, we used semi-structured interviews with patients and nurses, as well as observational field visits, to glean information while simultaneously build connections between our team and these individuals.[39]

One individual (CC) conducted in-person interviews in private spaces on or adjacent to the study wards; interviews were audio-recorded and transcribed verbatim by a professional service. The same team member (CC) observed the study wards for ~100 hours over 9 visits (May - June 2021), taking detailed field notes on overnight ward activity with particular attention to noise, light, interruptions, and factors emerging from interviews. All participants verbally consented to interviews, and all observed parties provided verbal assent prior to observation.

We used rapid assessment methods to summarize and synthesize interviews and observational field notes, grouping summary constructs under CFIR domains. CC, KP, AK, and PGL collectively summarized these data, reaching consensus around major themes by discussion. Based on insights from early interviews, we sought

© 2024 Catley CD, et al. *JAMA Network Open.*

quantitative information via the Verran and Snyder Halpern Sleep Scale (VSH)[6] and the electronic health record (EHR). During weekly site visits (concurrent with interviews) we invited patients to complete the VSH on tablet devices linked to Washington University's instance of Research Electronic Data Capture (REDCap; Vanderbilt University; Nashville, TN).[7] After completion of interviews and observations, we collected vital sign, laboratory, and medication administration data from the EHR (for the project wards), using the time stamps of these observations to calculate interruptions and sleep opportunity at baseline.

We integrated qualitative and quantitative data via building- and merging-based approaches to inform challenge mapping exercises.[8] We convened two groups of hospital leaders (including the Chief Nursing Officer and Patient Experience leads), nurse researchers with quality improvement expertise, hospitalist physicians, and clinical-translational sleep investigators for interactive challenge mapping and storyboarding sessions. Challenge mapping uses a series of structured questions to clarify and conceptualize complex problems prior to solution development.[9] Over two sessions in July and August 2021, the group summarized key findings via insight statements (succinct articulations of key learnings or priorities) and finalized problem statements to direct further work.

Intervention Development and Evaluation: Ideating, Prototyping, and Testing Solutions

Next, we led nurses and nurse leaders through two design workshops to ideate solutions and outline prototypes. Participants conceptualized potential interventions through structured interactive brainstorming exercises (e.g., rapidly generate as many ideas as possible based on insight statements, prioritizing quantity and outside-the-box ideas over depth or specificity).[5] In small groups, participants then co-developed hypothetical "bundles" of rest-improving interventions linked by thematic similarity and by expected resource requirements. A project manager trained in Agile methodology (KP) oversaw the iterative design of rest-promoting interventions from these hypothetical intervention bundles. Nursing staff, hospitalist physicians, and the project team collectively reviewed intervention design.

Quantitative Measures and Analysis

The co-primary outcomes were sleep opportunity and patient perceptions of nighttime quietness on the wards. We calculated sleep opportunity (a measure of process change, as fewer or clustered interruptions would yield more time available for continuous sleep) as the maximum time between overnight clinical interruptions (i.e., blood pressure measurements, blood draws, fingerstick glucose measurements, and scheduled medication administrations between 10 pm and 6 am).[10] We tabulated interventions based on EHR time-stamps and considered them to be unique if they occurred 15 minutes apart from each other (e.g., lab draw at 4:00am and blood pressure measurement at 4:20 am). Primarily, we compared the mean sleep opportunity per patient-night between the pre-intervention period and the final 2-week intervention sprint. Secondarily, we compared sleep opportunity between the pre-pilot period and each of the previous five sprints.

We quantified patient perceptions of nighttime quietness on the wards as the percentage of patients reporting that the wards were “always quiet” on routine HCAHPS surveys, which are made available to a random sample of adult patients discharged home after inpatient hospitalization of at least 24 hours. Across the entire hospital, BJH has historically invited approximately 650 discharged patients to take the HCAHPS survey each month, with completion rates ranging from 33-40%. Although organizational data tracking did not enable us to ascertain a denominator specific to this project, the study ward typically accounts for 3-5% of completed HCAHPS from the hospital.

We measured adoption as daily ward-level intervention uptake, reported by nursing managers. We assessed staff satisfaction with brief surveys. We quantified environmental noise as the number of times each unit’s nursing station experienced volumes above 35 decibels (via commercial YackerTracker devices).[11] Using EHR data, we calculated nightly clinical interruptions as the total number, per patient-night, of blood pressure measurements, blood draws for laboratory tests, fingerstick glucose measurements, and scheduled (but not “as needed”) medication administrations occurring between 10 pm and 6 am.

© 2024 Catley CD, et al. *JAMA Network Open.*

References

1. Damschroder LJ, Aron DC, Keith RE, Kirsh SR, Alexander JA, Lowery JC. Fostering implementation of health services research findings into practice: a consolidated framework for advancing implementation science. *Implement Sci*. 2009;4:50.

2. Lewis CC, Klasnja P, Powell BJ, et al. From Classification to Causality: Advancing Understanding of Mechanisms of Change in Implementation Science. *Front Public Health*. 2018;6:136.

3. Fuster M, Dimond E, Handley MA, et al. Evaluating the outcomes and implementation determinants of interventions co-developed using human-centered design to promote healthy eating in restaurants: an application of the consolidated framework for implementation research. *Front Public Health*. 2023;11:1150790.

4. Lyons PG, Chen V, Sekhar TC, et al. Clinician Perspectives on Barriers and Enablers to Implementing an Inpatient Oncology Early Warning System: A Mixed-Methods Study. *JCO Clin Cancer Inform*. 2023;7:e2200104.

5. Santhosh L, Rojas JC, Garcia B, Thomashow M, Lyons PG. Cocreating the ICU-PAUSE Tool for Intensive Care Unit-Ward Transitions. *ATS Sch*. 2022;3(2):312-323.

6. Shahid A, Wilkinson K, Marcu S, Shapiro CM. Verran and Snyder-Halpern Sleep Scale (VSH). In: Shahid A, Wilkinson K, Marcu S, Shapiro CM, eds. *STOP, THAT and One Hundred Other Sleep Scales*. Springer New York; 2012:397-398.

7. Harris PA, Taylor R, Thielke R, Payne J, Gonzalez N, Conde JG. Research electronic data capture (REDCap)--a metadata-driven methodology and workflow process for providing translational research informatics support. *J Biomed Inform*. 2009;42(2):377-381.

8. Fetters MD, Curry LA, Creswell JW. Achieving integration in mixed methods designs-principles and practices. *Health Serv Res*. 2013;48(6 Pt 2):2134-2156.

9. Basadur M. Reducing complexity in conceptual thinking using Challenge Mapping. *The International Journal of Creativity and Problem Solving*. 2003;13:5-27.

10. Najafi N, Robinson A, Pletcher MJ, Patel S. Effectiveness of an Analytics-Based Intervention for Reducing Sleep Interruption in Hospitalized Patients: A Randomized Clinical Trial. *JAMA Intern Med*. 2022;182(2):172-177.

11. Brown J, Fawzi W, Shah A, et al. Low stimulus environments: reducing noise levels in continuing care. *BMJ Qual Improv Rep*. 2016;5(1). doi:10.1136/bmjquality.u207447.w4214

© 2024 Catley CD, et al. *JAMA Network Open.*

Interview Guide

Introduction:

- Thank you for speaking with me. My name is _____. I work in the Healthcare Innovation Lab researching ways to improve the patient experience in the hospital. The goal of this project is to explore the experiences and perspectives of people who are hospitalized on the wards (outside the ICU) regarding sleep in the hospital.
- This interview will last about 15 minutes. We will ask you about your current and past experiences, and your perspectives on how sleep might be managed in an "ideal" hospital.
- What questions do you have for me before we begin?

Getting Acquainted:

- Please introduce yourself; tell me your name and why you are in the hospital.
- Thinking about your most recent day or two in the hospital, what does a typical day in the hospital look like for you?
- Thinking about your most recent night or two in the hospital, what does a typical night in the hospital look like for you?

Sleep:

- Overall, how would you describe your sleep in the hospital? How does this compare to your expectations?
- What things have helped you get a better night's sleep in the hospital?
- What things have prevented you from getting the kind of sleep you'd like to?
- What have you tried to do about these barriers to sleep? What's been helpful?
- Where should we focus our efforts to improve the nighttime environment in the hospital? In yor opinion, what would make the biggest difference?

Wrap-Up:

- Before we end our interview, what questions do you have for me? what else would you like to share about what we discussed?
- Thank you very much for your time and participation. If you have any subsequent questions or concerns for me, please contact me at _____.

© 2024 Catley CD, et al. *JAMA Network Open.*

	CODE	SUBCONSTRUCT NAME	DESCRIPTION
1	Innovation		
1.01	i_source	Source of innovation as internal or external	Perception by stakeholders of innovation or solution as being either internally or externally developed. May code I for internally developed and E for external
1.02	i_strength_quality	Strength and Quality of innovation	Stakeholders' perceptions of the quality and validity of evidence supporting the belief that the innovation will have desired outcomes. Include statements regarding awareness of evidence and the strength and quality of evidence, as well as the absence of evidence or a desire for different types of evidence.
1.03	i_adaptability	Adaptability	The degree to which an innovation can be adapted, tailored, refined, or reinvented to meet local needs. Include statements regarding the (in)ability to adapt the innovation to their context, e.g., complaints about the rigidity of the protocol.
1.04	i_complexity	Intervention complexity	Perceived feasibility and difficulty of implementation, reflected by duration, scope, radicalness, disruptiveness, centrality, intricacy and number of steps required to implement.

© 2024 Catley CD, et al. *JAMA Network Open.*

**1.05**	**i_relative_advantage**	**Relative advantage**	**Stakeholders' perception of the advantage [or disadvantage] of implementing a particular strategy over another. Include statements that demonstrate an innovation is better (or worse) than existing solutions. Exclude statements that demonstrate a strong need for the innovation and/or that the current situation is untenable and code to Tension for Change.**
**1.06**	**i_trialability**	**Trialiability**	**The ability to test intervention on a by unit basis, and to be able to reverse course (undo implementation) if warranted.**
**1.07**	**i_cost**	**Cost**	**Costs of the innovation and costs associated with implementing the innovation including investment, supply, and opportunity costs.**
**2**	**Recipients (Patients)**		
**2.01**	**r_motivation**	**Motivation**	**The reason(s) one has for acting or behaving in a particular way OR the general desire or willingness of someone to do something.**
**2.02**	**r_values**	**Values and beliefs**	**Individual or group principles or standards of behavior, ideas determine Rogers Theory of Diffusion Innovation, d to be "true", or what is determined to be important.**
**2.03**	**r_goals**	**Goals**	**The end state toward which an individual or group is striving.**
**2.04**	**r_skills_knowledge**	**Skills and knowledge**	**Abilities or proficiencies that are obtained through practice and the awareness of specific facts or strategies that they may be able to use**
**2.05**	**r_resources**	**Time, resources, support**	**Things or human resources that are used to achieve a goal, and anything that aids or assists in achieving a goal**

2.06	r_obstacles	Obstacles	Any obstacles to achieving desired outcome.
2.07	r_peer_pressure	Peer Pressure	Include statements about perceived pressure or motivation from other entities or organizations effecting patient behavior.
2.08	r_existing_stratg	Existing Strategies	The exisiting strategies or things that patients do to maintain or optimize sleep.
2.09	f_attributes	Facilitator attributes	Characteristics of facilitators, e.g., authenticity, openness, credibility, respected, responsive
2.10	r_authority	Power and authority	The percieved capacity or power of patients to influence events, behaviors, situations or people, even when others try to resist this influence.
3	Context (Local)		Individual ward
3.01	cl_leader_support	Formal and informal leadership support	The behaviors, attitudes and actions of leaders that reflect readiness or receptivity to a change.
3.02	cl_culture	Culture	Prevailing norms, values, and basic assumptions of a given organization, or “the way things are done around here”
3.03	cl_prior_innovation	Past experience of innovation and change	Prior experience with the introduction of something new.
3.04	cl_change_mechanisms	Mechanisms for embedding change	The process of incorporating a new thing or process such that it is fully implemented and consistently used in day-to-day work.
4	Context (Organizational)		Hospital-wide
4.01	co_priorities	Organizational priorities	Values and goals that guide the actions of an organization
4.02	co_leader_support	Senior leadership and management support	The behaviors, attitudes and actions of hospital leaders outside and above the ICU that reflect readiness or receptivity to a change.

© 2024 Catley CD, et al. *JAMA Network Open.*

4.03	co_culture	Culture	Prevailing norms, values, and basic assumptions of a given organization, or "the way things are done around here".
4.04	co_structure	Structure and systems	The social architecture, age, maturity, and size of an organization.
4.05	co_prior_innovation	History of innovation and change	Prior experience with the introduction of something new.
4.06	co_absorb_capacity	Absorptive capacity	The capacity for change, preparedness, and receptivity of involved individuals to an innovation, and the extent to which use of that innovation will be rewarded, supported, and expected within their organization.
4.07	cs_envi_stability	Environmental stability	The longevity and institutional memory of a space or a system.
4.08	co_learning_networks	Learning networks	Continuing education, collaboratives, or other organized efforts to enhance an individual or group's knowledge.
5	Context (System)		Entire health system
5.01	cs_priorities	System priorities	Values and goals that guide the actions within the US or global health system
5.02	cs_incentive_mandate	Incentives or mandates	Explicit or implicit prompts to encourage or enforce desired behavior
5.03	cs_redi_implement	Readiness for Implementation	Include statements regarding the general level of readiness for implementation.
6	Ideas		Ideas or input for potential intervention
6.01	id_idea	Ideas for Intervention	Any ideas or concepts put forth by interviewees about innovative changes to implement
6.02	id_light	Light	Indication that light is a problem when trying to sleep
6.03	id_physical	Physical Space	Indication that physical space is a problem when trying to sleep

**6.04**	**id_disruptions**	**Disruption**	**Indication that being woken up by healthcare team is a problem when trying to sleep**
**6.05**	**id_noise**	**Noise**	**Indication that noise is a problem when trying to sleep**

© 2024 Catley CD, et al. *JAMA Network Open.*

Sleep education handouts

INNOVATION LAB & UNIT 5500

Rest Is Best

Sleep is essential for a person's health. It is the foundation of our physical well-being, mental resilience, and immune system support. In short, being well-rested helps us to show up as the best version of ourselves.

- **1. Sleep is Restorative**
  When you sleep, you allow your body to repair and rebuild. During this time, the body can clear debris from the lymphatic system, which boosts the immune system. When you sleep, important processes happen, including muscle repair, protein synthesis, tissue growth, and hormone release.
- **2. Sleep Reduces Stress**
  Sleep improves concentration, regulates mood, and sharpens judgement and decision-making. A lack of sleep reduces mental clarity, as well as our ability to cope with stressful situations. This is due in part to the impact of chronically high levels of cortisol.
- **3. Sleep Raises Cortisol Levels**
  High cortisol levels are important in the short term, stimulating alertness, raising heart rate and bp, but over time can cause systemic inflammation and disrupt our hormonal balance. Cortisol levels fall in the evening hours as part of sleep. When we put off sleep, cortisol levels remain high and interfere with the release of melatonin which is essential for regulating sleep cycles.
- **4. Sleep Improves Memory**
  Sleep serves as an opportunity for the mind to process all the stimuli that we have taken in while we are awake and triggers changes in the brain that strengthen neural connections helping us to form memories. Sleep is also vital to memory consolidation which is important for learning new information.
- **5. Sleep Helps Maintain Healthy Weight**
  When you are sleep deprived your body alters the hormones that regulate hunger and appetite: leptin & ghrelin. Sleep deprivation can also activate the endocannabinoid system in our brain which increases hunger and appetite, especially for junk food.
- **6. Sleep May Prevent Illnesses**
  Lack of sleep has been linked to chronic diseases such as diabetes, heart disease, and obesity. Sleep deprivation makes you more prone to illness because your immune system isn't performing to its peak. And you often don't feel these negative effects until it's too late.
- **7. Sleep is Important for Mental Health**
  Lack of sleep contributes to the formation of new mental health problems and to the maintenance of existing ones. Disrupted sleep is commonly seen as both a symptom and consequence of mental health disorders, most commonly insomnia.
- **8. The Most Common Sleep Problem**
  Insomnia, sustained difficulty falling or staying asleep, is often associated with poor mental health. It has been found to worsen mental health disorders, especially paranoia and hallucinations. Symptoms include not feeling well-rested after a night's sleep, daytime tiredness, irritability, depression, anxiety, increased errors or accidents, and ongoing worries about sleep.
- **9. How to Improve Sleep**
  Establish a bedtime and stick to it; maintain comfortable temperature settings and low light levels in the bedroom; limit screentime on television, phones, etc.; abstain from caffeine, alcohol, and large meals leading up to bedtime; refrain from tobacco use; exercise during the day to help wind down for sleep.
- **10. Sources**
  Verywellhealth.com: The Importance of Sleep; 10 Top Health Benefits of Sleep

© 2024 Catley CD, et al. *JAMA Network Open.*

INNOVATION LAB & UNIT 5500

Rest Is Best:

Reduce Unnecessary Interruptions

Sleep is essential for a person's health. It is the foundation of our physical well-being, mental resilience, and immune system support. In short, being well-rested helps us to show up as the best version of ourselves.

Below are a few reminders to help enhance our patients' sleep and reduce unnecessary additional interruptions.

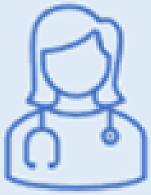

Lab Draws & Vital Signs

Cluster care at night to reduce additional interruptions to patients' sleep. Get lab draws when collecting vitals and giving medications, when applicable. Look at Physician's order times and make sure they make sense for the patient.

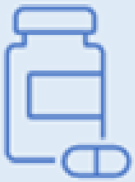

Sleep Medications

Proactively prescribe/distribute sleep aids. This allows patients to get the sleep they need, when they need it.

© 2024 Catley CD, et al. *JAMA Network Open.*

INNOVATION LAB & UNIT 5500

Rest Is Best: Getting Quality Sleep

Sleep is essential for a person's health. It is the foundation of our physical well-being, mental resilience, and immune system support. In short, being well-rested helps us to show up as the best version of ourselves.

Below are a few reminders to help enhance our patients' sleep.

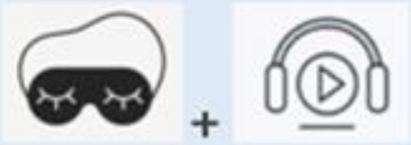

Offer a Sleep Kit & Sound Machine to Each Patient

Sleep kits, which come with an eye mask and ear plugs, will be included in each patient's admission kit. Be sure to point this out to them.

At shift handoff, offer each patient a sound machine to help them sleep better. Noise machines have multiple sound and timer options. Mark down which room number received a noise machine on the tracker on the huddle board.

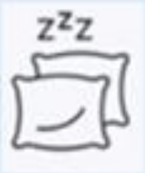

Ask About Personal Sleeping Preferences

Ask patients if they have any particular sleeping preferences, such as:

- Sleeps with 2 pillows
- Keeps a nightlight on
- Needs a fan on to stay cool

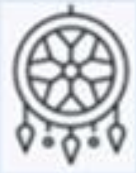

Additional Activities

To prevent boredom, ask patients if they want something out of the activity cabinet to keep their mind occupied. You can also order a consult from Art Therapist, Sarah Colby.

© 2024 Catley CD, et al. *JAMA Network Open.*

Staff satisfaction survey instruments

Sprint 1

Question 1: On a scale of 1 to 5, how would you rate the helpfulness "Rest is Best" flyer?

Question 2: On a scale of 1 to 5, how beneficial did you find the established quiet hours for you &/or your patients?

Sprint 2

Question 1: During your shifts over the last 2 weeks, how often did you participate in clustering care for your patients?

Question 2: During your shifts over the last 2 weeks, how often did you wear your red light keychain?

Sprint 3

Question 1: During your shifts over the last 2 weeks, how often did you participate in clustering care for your patients?

Sprint 4

Question 1: How beneficial did you find the overall practice of helping patients improve their sleep

© 2024 Catley CD, et al. *JAMA Network Open.*

eFigure 1. Logic Model.

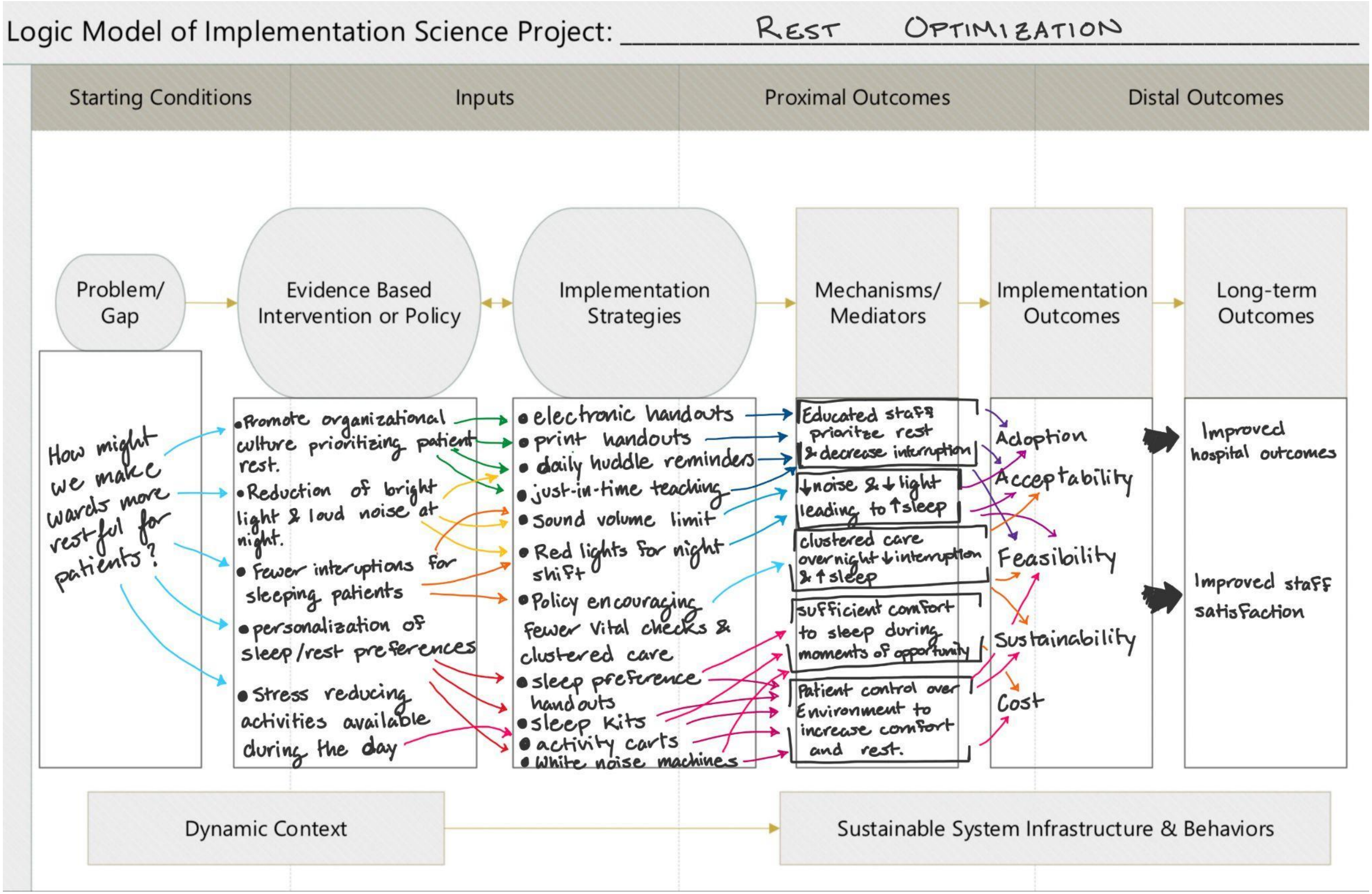

© 2024 Catley CD, et al. *JAMA Network Open.*

eFigure 2. Challenge map.

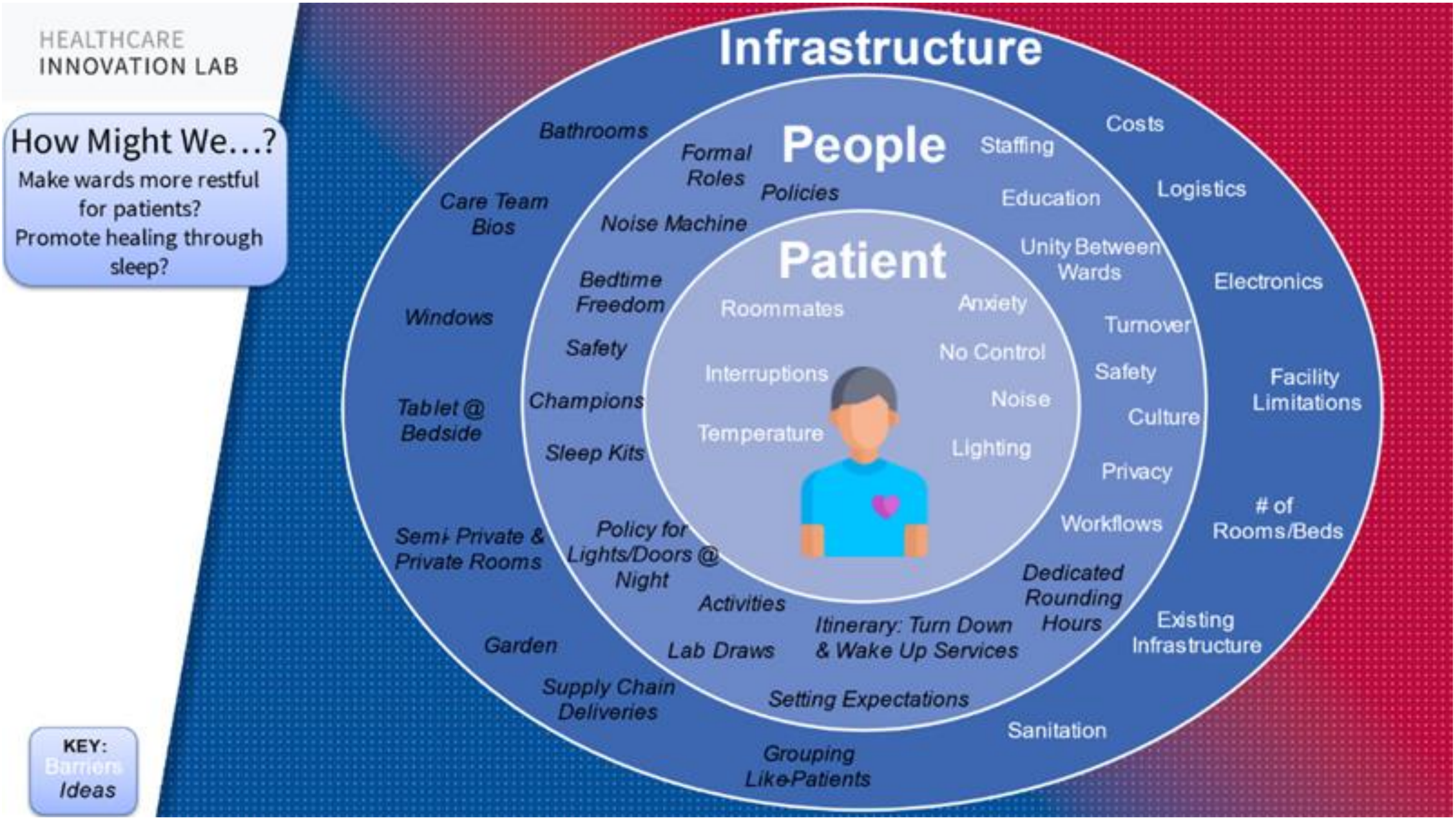

© 2024 Catley CD, et al. *JAMA Network Open.*

eFigure 3. Intervention Bundle concepts

© 2024 Catley CD, et al. *JAMA Network Open.*

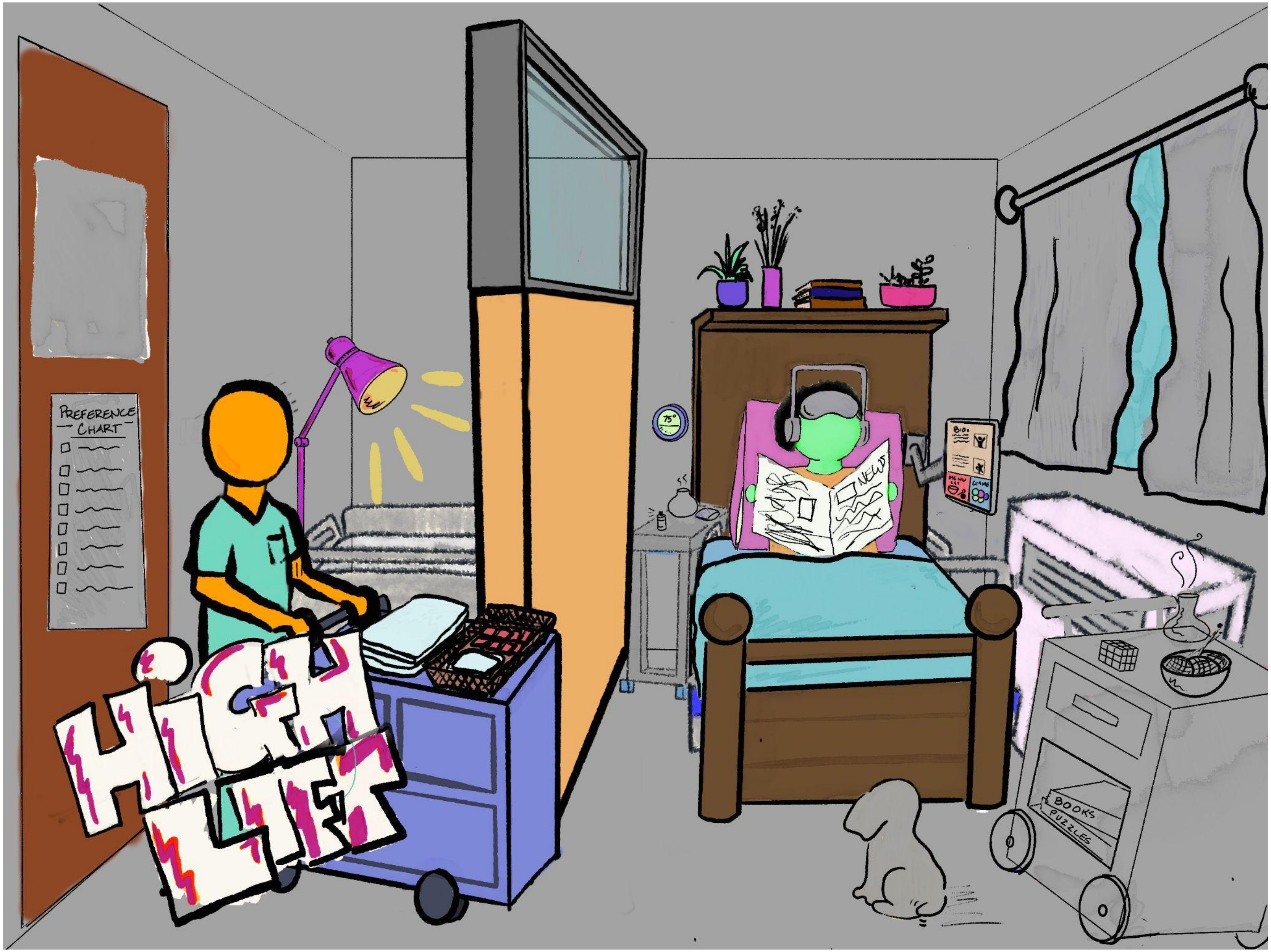

© 2024 Catley CD, et al. *JAMA Network Open.*

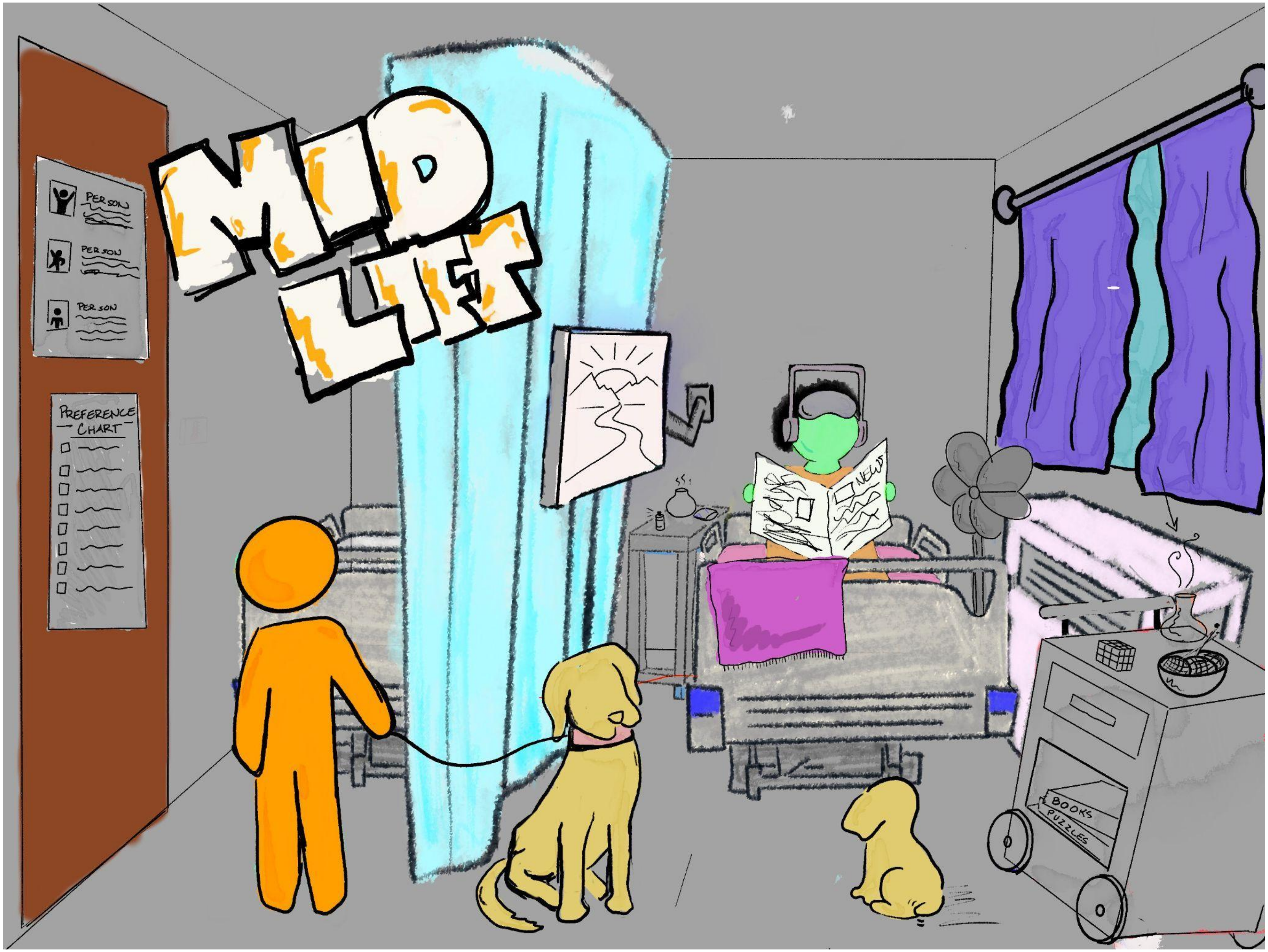

© 2024 Catley CD, et al. *JAMA Network Open.*

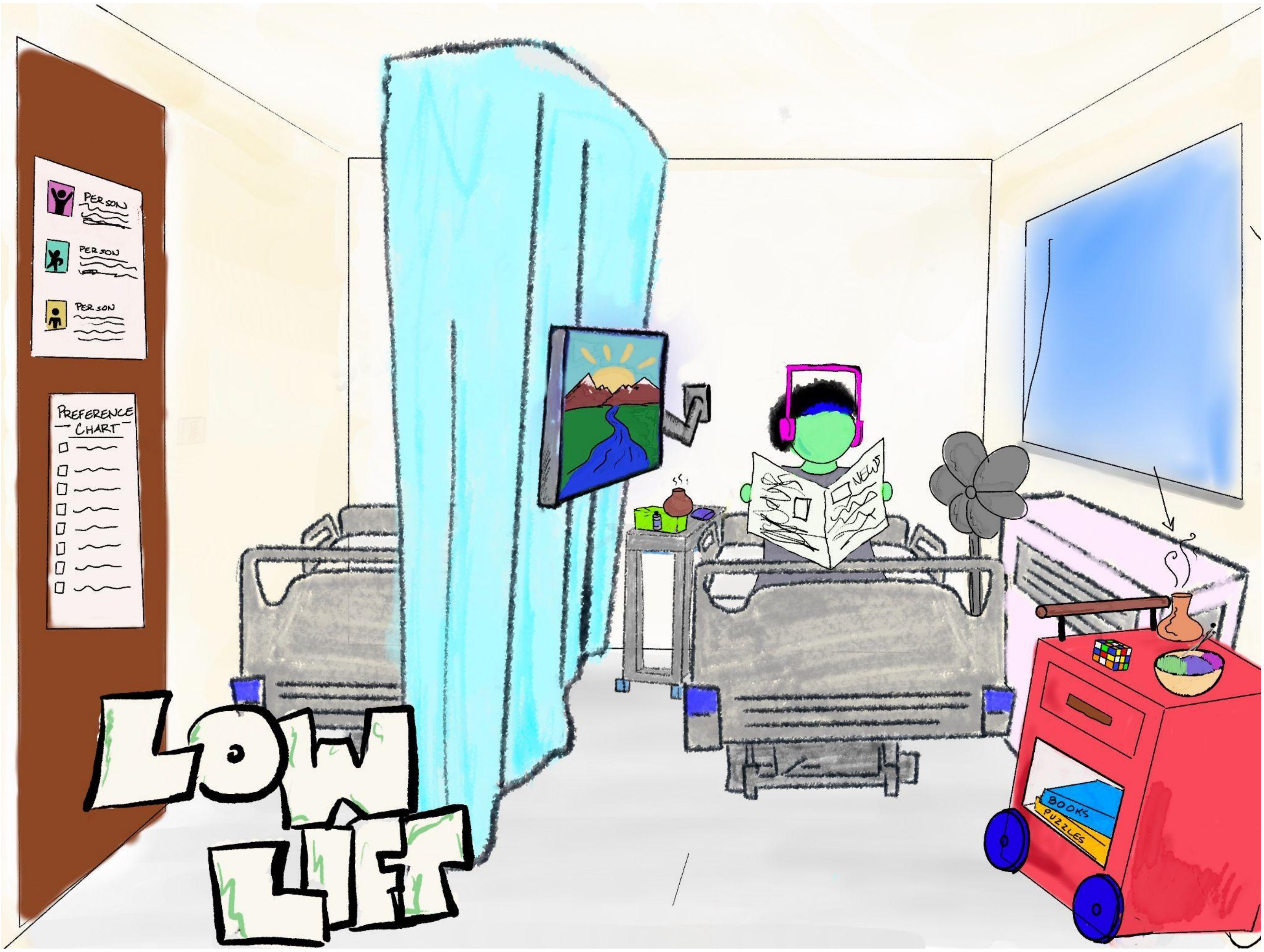

© 2024 Catley CD, et al. *JAMA Network Open.*

eTable 1. Determinants of restfulness on the wards and their relationship to candidate interventions.

Domain	Determinants	Example	Intervention Idea(s)
Inner Setting			
Physical Infrastructure	Hospital construction (noise)	"Right now they're doing construction, so it's been very noisy and very hard to get sleep in the hospital right now." (Patient 7, Ward B)	• Explicit quiet hours<br>• White noise machines<br>• Sleep kits with ear plugs
	Double-occupancy rooms (noise)	"They still have double rooms. So if one patient's trying to sleep, they're getting usually disturbed by the other patient" (Nurse 2, Ward A)	• Eliminate double-occupancy rooms<br>• Higher-quality room dividers<br>• Default use of TV timers (for roommate's TV)<br>• Nightly request for phones on vibrate (for roommate's device)
	Ward layout (noise)	"These rooms are close to the nurses' station, so it's always louder. Typically, the patients that are closer to the nurses' station are patients that need to be monitored more closely, whether that would be they're just forgetful and try to get up, they're sicker, they need more attentive care" (Nurse 5, Ward A)	• Explicit quiet hours<br>• White noise machines<br>• Sleep kits with ear plugs<br>• Default use of TV timers<br>• Ward-level decibel tracker competition
	Lighting	"Sometimes the, the lighting [is bright] and they forget to close the door. And it-- you know, it shines in your eyes." (Patient 2, Ward B)	• Ensure lights off at bedtime<br>• Ensure door closed at bedtime<br>• Clip-on red light flashlights<br>• Sleep kits with eye mask and ear plugs<br>• Preference checklist: temp, light, routine
	Temperature	"At least try to get them comfortable, temperature and bed-- sometimes that helps" (Nurse 2, Ward A)	• Fans available for each patient<br>• Increased blanket availability<br>• More comfortable linens<br>• More comfortable gowns<br>• Higher-quality pillows<br>• Smarthome hospital rooms<br>• Preference checklist: temp, light, routine
IT Infrastructure	Medical Devices	"Patients that are on continuous fluids-- if their IV is beeping-- or even if they're on antibiotics, but that also wakes them up. So they get woken	• "Smarter" IV pumps

© 2024 Catley CD, et al. *JAMA Network Open.*

		up to get the antibiotic started, and then it beeps, and then they get woken up again 30 minutes to an hour later because it's finished, so that interrupts their sleep as well… [I have heard of an idea where] instead of beeping out loud in the patient's room [the IV pump would] trigger something to go to the nurse's phone, which would be good. That way it doesn't wake up the patient or the roommate. So I thought that would be cool, but, again, that's a lot of equipment that needs to be bought and tried." (Nurse 8, Ward A)	
	Electronic health record		• Sleep-promoting EHR order defaults (e.g., routine medication start times, overall and nighttime vital sign frequency, timing of routine lab draws)
Work Infrastructure	Nursing workflows	"I watch TV, fall asleep, nurse come wake me up. Fall back to sleep. Nurse come back and wake me up." (Patient 4, Ward B)<br><br>"They'll come to wake you up to do assessments, dressing changes, wound care, lab draws, meds. Nurse rounding anyways every hour, just to make sure everybody's okay." (Nurse 5, Ward A)	• Restricted times for routine lab draws by policy<br>• Restricted times for routine medications by policy<br>• Routine measurement/reporting of ward-level rates of abnormal nocturnal vital signs (i.e., for context when considering reducing overnight interruptions for individual patients)<br>• Daily "patient routine" templates (e.g., on whiteboard in room)<br>• Educate staff on value of sleep<br>• Explicit quiet hours (signage, expectations)
	Competing priorities	If we can't get a person's labs, then we have to have multiple people go in and attempt to stick whenever they have time. So it could be not until 1 or 2 o'clock in the morning that another nurse has the chance to go in and try to stick that patient. (Nurse 8, Ward A)	• More staffing<br>• Educate staff on value of sleep<br>• Sleep on problem list: standard ask
	Laboratory workflows		• Restricted times for routine lab draws and tests by policy<br>• Sleep-promoting EHR order defaults (e.g., routine medication start times,

© 2024 Catley CD, et al. *JAMA Network Open.*

			overall and nighttime vital sign frequency, timing of routine lab draws)
	Patient transfers	"But if the patient is-- for example, just got back from a procedure that day or just that evening, vitals have to be done, assessments have to be done just to make sure that that patient stays stable, which means you're not going to get to sleep." (Nurse 5, Ward A)	● Daily "patient routine" templates (e.g., on whiteboard in room)<br>● Restricted times for routine lab draws and tests by policy<br>● Sleep-promoting EHR order defaults (e.g., routine medication start times, overall and nighttime vital sign frequency, timing of routine lab draws)
	Automated care routines	"When you have a Q12H antibiotic and it gets delayed during the day for a procedure, then it gets given at 3PM, then you have to get it again at 3AM-- now you're waking that patient up at 3AM to [give them] antibiotics" (Nurse 8, Ward A)	● Sleep-promoting EHR order defaults (e.g., routine medication start times, overall and nighttime vital sign frequency, timing of routine lab draws)<br>● Patient education: available options/interventions (e.g., might be able to request less frequent overnight vital signs)
Compatibility		"If it's something that can wait till 6:00 am, we can try to cram it in [to avoid nocturnal interruptions], but we can't do 12 hours of work in four hours." (Nurse 1, Ward A)<br><br>"I wish the workload can be reduced so that we can give our patients that extra that they deserve. Because sometimes when you have too much to do, you are not able to give that extra to the patient because you find yourself, you are in the patient's room, your heart is in another room because you actually want to serve everybody. You don't want to rob Peter to pay Paul. You don't want to give one person care at the expense of another… I'm a registered nurse, but I can make use of some ancillary staff that can help my work a lot easier, like the techs. Those that can assist with patients' care, like the secretary, sometimes we do have, and sometimes we don't. So you find	● More staffing<br>● Educate staff on value of sleep<br>● Patient education: available options/interventions (e.g., might be able to request less frequent overnight vital signs)<br>● Sleep on problem list (i.e., emphasize rest as a priority to be considered alongside other medical problems)<br>● Explicit quiet hours (signage, expectations)

© 2024 Catley CD, et al. *JAMA Network Open.*

		out that you are doing the work of a nurse, at the same time, you are answering phone calls, you are drawing blood, you are doing a lot. And even up to taking care of trash… We are taking out trays. We are serving the patients food. Those extras. If we can have those taken off, then we'll be more focused on the patients' medical care. Then those other things, which are also very important for the patients' comfort, if we have other people helping to do that, that can also help a lot to reduce the workload. And of course, the nurse-patient ratio. It can also help a lot. If we have good nurse-patient ratio, that will help. (Nurse 10, Ward B)	
Relative Priority	Routine care	"There's always noise, like I said, from pumps beeping, or us having to wake patients up and do stuff, so it is hard to get a full night's rest here probably, but a lot of patients don't get the rest that they need just because they're always having to do something, so it's hard because you need adequate rest to heal, but we also need to do other stuff to help you get better, too." (Nurse 6, Ward A)	● Patient education: available options/interventions (e.g., might be able to request less frequent overnight vital signs)<br>● Educate staff on value of sleep<br>● Better handoff: Educate on meds/practices<br>● Sleep-promoting EHR order defaults (i.e., change what "routine" means)<br>● Sleep on problem list<br>● Explicit quiet hours (signage, expectations)
	Timely and complete handoff	"Then at about 4:00 AM, you want to start with your vital signs again. Because I like to make sure of the patient I'm giving to the next shift. So whether they are due for vital signs [or not]… you start off with your vital signs, and then you give some medications that are due. For those who are due for medication at 6:00, you want to go into their room when it's almost 5:00 so that you just do the vital signs, give your medication, flush their IVs, and that is it. Then you make the patient comfortable, check the continent pad to be sure they are clean, if not, you want to change them for the next shift." (Nurse 10, Ward	● Educate staff on value of sleep<br>● Better handoff: Educate on meds/practices

© 2024 Catley CD, et al. *JAMA Network Open.*

		B)	
	Nurse self-care	“We're really loud out here in the hall, especially when you're closer to the nurses' station it can be really, really loud, which is hard because sometimes at night when you're trying to stay awake, being quiet isn't super easy. Well, it's easy to be quiet, which means you're going to fall asleep, so got to stay talking, got to stay up and be chipper.” (Nurse 5, Ward A)	• Educate staff on value of sleep<br>• Better handoff: Educate on meds/practices<br>• Sleep on problem list: standard ask<br>• Explicit quiet hours (signage, expectations)
Mission Alignment		“It's kind of the expectation that we do provide 24-hour care, but understanding that sleep is an important part of recovery, it's hard to balance those things. And so I think it should be better, but I don't know how to fix that.” (Nurse 8, Ward A)<br><br>“And when you're going in there, you be like, ‘Look this is a hospital, and we're here to make you better, so I'm going to be in here a lot more than you will want me to be in here.’ That's what we will tell the patients, but I could see that's why they wouldn't get a good night's sleep” (Nurse 5, Ward A)<br><br>Well, on our end, what we should focus on is our patients' comfort. We shouldn't just be dictators like, "Look, we have this due at this time, let's--" Mm-mm, it should be a collaboration. We should involve patients in their care. "Oh, how do you want it? What time do you want it?" Let the patient also be on top of their care. And when they have the sense of being in charge, they cooperate more, and then there is no tension anywhere, and everybody is relaxed. The caregiver is relaxed giving care, you are not tensed up. And of course, the patient is relaxed receiving care and is not tensed up. So I think from us to the patient, we	• Educate staff on value of sleep<br>• Better handoff: Educate on meds/practices<br>• Sleep on problem list: standard ask<br>• Explicit quiet hours (signage, expectations)

© 2024 Catley CD, et al. *JAMA Network Open.*

		should allow that collaboration. We should allow that-- patients should be involved in their care. It will really help. (Nurse 10, Ward B)	
Available resources		Last night, they gave me some Tylenol and Benadryl. It helped some but still only get a few hours a night.<br><br>"I mean, we give our patients, think what they call, a quiet night kit or something that has like an eye mask and ear plugs. But honestly, that's throwing a Band-Aid on a hemorrhaging wound." (Nurse 1, Ward A)<br><br>"We have fans here in the hospital, but that's not going to mask your neighbor's IV pump going off every five minutes" (Nurse 5, Ward A)	• Daily "turndown service" / sleep concierge<br>• Patient education: Sleep importance, available options/interventions<br>• Preference checklist: temp, light, routine<br>• Make simple interventions - sleep kits, white noise machines, relaxation activities, etc. - available by default rather than "need to ask"<br>• Daily "patient routine" templates (e.g., on whiteboard in room - to allow patients to better anticipate their own potential needs)
<u>Outer Setting</u>			
Local Attitudes	Cultural perceptions of hospital monitoring (necessary and protective) and sleep (a potential impediment to care rather than an essential part of care)	"Ultimately, the care that you need is a higher priority than sleep" (Nurse 5, Ward A)	• Educate staff on value of sleep<br>• Better handoff: Educate on meds/practices<br>• Preference checklist: temp, light, routine<br>• Explicit quiet hours (signage, expectations)<br>• Daily "patient routine" templates (e.g., on whiteboard in room)<br>• Ward-level decibel tracker competition<br>• Restricted times for clinical rounding by policy<br>• Restricted times for routine lab draws and tests by policy<br>• Restricted times for routine medications by policy<br>• Sleep on problem list: standard ask<br>• Routine measurement/reporting of ward-level rates of abnormal nocturnal vital signs<br>• (all serve to "normalize" rest promotion as part of culture)
Policies and Laws<br>External Pressures	Perceptions of hospital care as compliance-driven may lead to repressive attitudes and/or risk-avoidant behaviors.	"Even if the patient says, 'Turn off all my lights,' what I do is I'll turn on the bathroom light and I'll shut the door partway so there's enough light that they can see. I don't want anybody tripping on my shift. Too much paperwork. (Nurse 9, Ward B)	

© 2024 Catley CD, et al. *JAMA Network Open.*

<u>Individuals</u>			
Innovation Deliverers	Attitudes and Relative Priority (as above)	"Sometimes we maybe talk a little bit outside patients' care. So when we do so, we can use low tones because sometimes when we'll talk about things, maybe we're talking about sports, fashion, maybe other things." (Nurse 10, Ward B)	• Educate staff on value of sleep<br>• Better handoff: Educate on meds/practices<br>• Ward-level decibel tracker competition<br>•
Recipients	Patients' ability to (and awareness of the imperative to) self-advocate	"We have a patient now that's like, 'Don't wake me up for 5:00 AM vitals. I'm going to be sleeping.' And we're like, 'Okay.' If you tell us in advance, we'll just let the doctor know you've refused your morning vitals. But if we don't get that verbal cue to do that, then we have to come in and do your vitals." (Nurse 8, Ward A)<br><br>If the doctors are okay with writing do not disturb orders between 12:00 and 5:00 or 6:00? You don't really want to go all the way to seven because there are some things you got to get done before day shift between the hours of five and seven. But if they can-- if they cannot have orders for us, at least on our stable patients, to do not disturb between eleven and five? I feel like that's doable, but I think the patients that ask for that will get it. It's just most patients don't ask for it. (Nurse 1, Ward A)<br><br>"if they request that we don't wake them up between certain times, that person is usually alert and oriented and can tell you" (Nurse 9, "Ward B)	• Patient education: Sleep importance, available options/interventions<br>• Preference checklist: temp, light, routine<br>• Daily "patient routine" templates (e.g., on whiteboard in room)<br>• Daily "turndown service" / sleep concierge<br>• Care team biographical information handouts (i.e., so patients are more familiar with whom they might ask)
	Boredom	Watch TV, sleep. Sister come, Mama come. Nurse in and out. And that's it." (Patient 4, Ward B)<br><br>"Goes by sometimes slow, sometimes fast" (Patient 2, Ward B)	• Increase recreational therapist presence<br>• Increased activity cart availability<br>• Daily newspaper availability<br>• Meditation mobile application<br>• Default use of TV timers (i.e., to improve nocturnal sleep hygiene)<br>• Smarthome hospital rooms

© 2024 Catley CD, et al. *JAMA Network Open.*

		"Uh, I sleep, mostly (during the day)" (Patient 2, Ward B)<br><br>"Long and boring." (Patient 8, Ward B)	
	Anxiety	"I get anxious." (Patient 6, Ward B)<br><br>"[Patients are often] anxious and they'll say, 'I'm really having a hard time sleeping because this is different environment'" (Nurse 9, Ward B)<br><br>"Oh, I'll say sleep at home is, is so different... At the house is more activities to do. You know, like watching TV, go to the backyard, or do stuff like that." (Patient 8, Ward B)	• Aromatherapy<br>• Increase recreational therapist presence<br>• Routine therapy animal visits<br>• Daily newspaper availability<br>• Default soothing TV channel<br>• Meditation mobile application<br>• Daily "turndown service" / sleep concierge<br>• Care team biographical information handouts (i.e., for more familiarity)
	Variation in patient experience	"The hospital is a safer place for me" (Patient 9, Ward not recorded)	• Preference checklist: temp, light, routine

© 2024 Catley CD, et al. *JAMA Network Open.*

eTable 2. Patient Characteristics and Ward-Level Outcomes.

	Pre-Intervention, n = 217	Intervention 1, n = 86	Intervention 2, n = 74	Intervention 3, n =229	Post-Study, n = 65	P-value
**Age, mean (SD)**	61 (17)	62 (14)	59 (16)	60 (16)	56 (17)	0.34
**Female, n (%)**	133 (61%)	39 (45%)	21 (28%)	113 (49%)	30 (46%)	0.04
**Comorbidities, n (%)**						
**CHF**	59 (27%)	31 (36%)	19 (26%)	65 (28%)	23 (35%)	0.02
**COPD**	30 (14%)	13 (15%)	8 (11%)	49 (21%)	8 (12%)	0.18
**OSA**	16 (7.4%)	4 (4.7%)	9 (12%)	18 (7.9%)	9 (14%)	0.26
**Cancer**	26 (12%)	8 (9.3%)	5 (6.8%)	30 (13%)	8 (12%)	0.52
**CKD**	73 (34%)	16 (19%)	12 (16%)	43 (19%)	19 (29%)	<0.01
**Length of Stay, days, median (IQR)**	5.1 (2.8-9.7)	4.3 (2.8-8.6)	6.2 (3.9-10.8)	5.8 (3.4-11.8)	6.1 (3.8-13.4)	0.40
**Hospital Mortality, n (%)**	1 (<1%)	2 (2.3%)	2 (2.7%)	6 (2.6%)	3 (4.6%)	0.02

SD, standard deviation; CHF, congestive heart failure; COPD, chronic obstructive pulmonary disease; OSA, obstructive sleep apnea; CKD, chronic kidney disease; IQR, interquartile range

© 2024 Catley CD, et al. *JAMA Network Open.*

eTable 3. Study Outcomes Across Interventions.

	Pre-Intervention, n(patients) = 217, n(patient-nights) = 981	Intervention 1, n(patients) = 86. n(patient-nights) = 266	Intervention 2, n(patients) = 74, n(patient-nights) = 328	Intervention 3, n(patients) = 229, n(patient-nights) = 1179	Post-Study, n(patients) = 65, n(patient-nights) = 237	P-value
**Co-Primary Outcomes**						
Patients rating hospital wards as "always quiet",[a] n (%)	24/47 (51%)	NA	NA	10/15 (67%)	6/7 (86%)	0.06
Sleep opportunity per patient-night,[b] hours, mean (SD)	4.94 (1.86)	4.84 (1.55)	4.78 (1.59)	4.75 (1.56)	5.10 (1.65)	0.01
**Secondary Outcomes**						
Clinical interruptions per patient-night,[c] mean (SD)	2.69 (1.95)	2.95 (1.85)	2.97 (2.07)	2.75 (1.74)	2.78 (2.02)	0.09
Excessive noise events per 100 patient-nights,[d] mean (SD)	0.65 (1.3)	0.0 (0.0)	0.0 (0.0)	0.0 (0.0)	0.0 (0.0)	0.02

© 2024 Catley CD, et al. *JAMA Network Open.*

[a]Calculated as n (%) rating hospital wards as "always quiet" on post-hospitalization HCAHPS
[b]Calculated as longest time between EHR-measured interruptions, 10pm-6am
[c]Calculated as unique recordings of blood pressure, fingerstick blood glucose, laboratory blood tests, and scheduled medication administrations
[d]Calculated as number of recordings > 35 dB per night
SD, standard deviation; HCAHPS, Hospital Consumer Assessment of Healthcare Providers and Systems

© 2024 Catley CD, et al. *JAMA Network Open.*

eTable 4. Clinical Interruptions Across Interventions.

	Pre-Intervention, n(patient-nights) = 981	Intervention 1, n(patient-nights) = 266	Intervention 2, n(patient-nights) = 328	Intervention 3, n(patient-nights) = 1179	Post-Study, n(patient-nights) = 237	P-value
Blood pressure measurements per patient-night, mean (SD)	1.71 (1.34)	1.40 (1.09)	1.57 (1.08)	1.47 (1.10)	1.66 (1.80)	<0.01
Fingerstick blood glucose measurements per patient-night, mean (SD)	1.76 (0.98)	1.94 (1.57)	1.73 (0.79)	1.73 (0.92)	1.72 (0.80)	0.73
Laboratory blood draws per patient-night, mean (SD)	1.27 (0.56)	1.20 (0.54)	1.20 (0.54)	1.27 (0.64)	1.26 (0.60)	0.51
Medication administrations per patient-night, mean (SD)	1.80 (0.98)	1.75 (0.86)	2.01 (1.13)	1.74 (1.04)	1.79 (1.03)	0.03
SD, standard deviation						

© 2024 Catley CD, et al. *JAMA Network Open.*
