# Supplementary material for: Redesigning the Hospital Environment to Improve Restfulness
Source: JAMA Netw Open. 2024 Dec 4;7(12):e2447790. doi: 10.1001/jamanetworkopen.2024.47790 (PMC11618460; doi:10.1001/jamanetworkopen.2024.47790)
Supplement: Supplement 2. — Data Sharing Statement [file jamanetwopen-e2447790-s002.pdf]

Data Sharing Statement

Catley. Redesigning the Hospital Environment to Improve Restfulness. *JAMA Netw Open*. Published December 04, 2024. doi:10.1001/jamanetworkopen.2024.47790

Data
Data available: No
